# Hcp Proteins of the Type VI Secretion System Promote Avian Pathogenic *E. coli* DE205B (O2:K1) to Induce Meningitis in Rats

**DOI:** 10.3390/life12091353

**Published:** 2022-08-31

**Authors:** Xuhang Wang, Yu Sun, Dinesh Subedi, Qianwen Gong, Haosheng Huang, Jin Li, Yuxin Wang, Jianluan Ren

**Affiliations:** 1 MOE Joint International Research Laboratory of Animal Health and Food Safety, Key Laboratory of Animal Bacteriology, Ministry of Agriculture, College of Veterinary Medicine, Nanjing Agricultural University, Nanjing 210095, China; 2 School of Biological Sciences, Monash University, Clayton, VIC 3800, Australia

**Keywords:** meningitis, APEC, zoonotic, Type VI secretion system, pathogenicity, brain microvascular endothelial cells

## Abstract

Avian pathogenic *Escherichia coli* (APEC) is an important extra-intestinal pathogenic *E. coli* (ExPEC), which often causes systemic infection in poultry and causes great economic loss to the breeding industry. In addition, as a major source of human ExPEC infection, the potential zoonotic risk of APEC has been an ongoing concern. Previous studies have pointed out that APEC is a potential zoonotic pathogen, which has high homology with human pathogenic *E. coli* such as uro-pathogenic *E. coli* (UPEC) and neonatal meningitis *E. coli* (NMEC), shares multiple virulence factors and can cause mammalian diseases. Previous studies have reported that O18 and O78 could cause different degrees of meningitis in neonatal rats, and different serotypes had different degrees of zoonotic risk. Here, we compared APEC DE205B (O2:K1) with NMEC RS218 (O18:K1:H7) by phylogenetic analysis and virulence gene identification to analyze the potential risk of DE205B in zoonotic diseases. We found that DE205B possessed a variety of virulence factors associated with meningitis and, through phylogenetic analysis, had high homology with RS218. DE205B could colonize the cerebrospinal fluid (CSF) of rats, and cause meningitis and nerve damage. Symptoms and pathological changes in the brain were similar to RS218. In addition, we found that DE205B had a complete T6SS, of which Hcp protein was its important structural protein. Hcp1 induced cytoskeleton rearrangement in human brain microvascular endothelial cells (HBMECs), and Hcp2 was mainly involved in the invasion of DE205B in vitro. In the meningitis model of rats, deletion of *hcp2* gene reduced survival in the blood and the brain invasiveness of DE205B. Compared with WT group, *Δhcp2* group induced lower inflammation and neutrophils infiltration in brain tissue, alleviating the process of meningitis. Together, these results suggested that APEC DE205B had close genetic similarities to NMEC RS218, and a similar mechanism in causing meningitis and being a risk for zoonosis. This APEC serotype provided a basis for zoonotic research.

## 1. Introduction

Bacterial meningitis is a common and serious disease of the central nervous system, and is an important cause of morbidity and death in newborns [1,2]. Despite advances in antimicrobial treatment and prevention, the annual case fatality rate for meningitis remains between 15% and 40%, with approximately 50% of survivors suffering from neurological sequelae and close to 100% of untreated bacterial meningitis patients dying [3,4]. *Escherichia coli* (*E. coli*) is the leading cause of neonatal meningitis, second only to type B Streptococcus. In clinical isolates, most meningitis-associated *E. coli* (NMEC) have a K1 capsule [5].

The adhesion and invasion of BMECs by *E. coli* is a key step for *E. coli* in penetrating the blood–brain barrier (BBB) and entering the brain tissue to mediate *E. coli* meningitis [4]. RS218 strain is the classic strain in NMEC [6,7]. *CUS-3*, also referred to as RDI 12, is one of the virulence islands of RS218. *CUS-3* is a prophage island unique to the RS218 strain [8]. IbeA, OmpA and FimH are involved in RS218 induced meningitis [9,10]. The K1 capsule of RS218 contributes to the survival of bacteria in blood, macrophages and human brain microvascular endothelial cells (HBMECs), and the OmpA protein encoded by RS218 promotes the survival of *E. coli* K1 in neutrophils, macrophages and HBMECs [11,12,13,14,15]. Knockout of neutrophils or macrophages in neonatal mice can prevent the occurrence of *E. coli* meningitis [16,17], and the OmpA protein can also bind to the gp96 receptor on the surface of HBMECs to activate the FAK signaling pathway and mediate the destruction of the blood–brain barrier (BBB) [18,19]. In addition, cytotoxicity factor 1 (CNF1) and IbeA bind to the 37LRP receptor and Caspr1 receptor on the surface of HBMECs, respectively, to promote cytoskeletal rearrangement and bacterial adhesion and invasion [20,21]. The Type VI secretory system (T6SS) is a novel secretory system first discovered in *Vibrio cholerae* and found in many Gram-negative bacteria [22]. Studies have shown that T6SS played an important role in the adhesion and invasion of RS218 strains in HBMECs. Hcp was considered the structural protein of a functional T6SS. Hcp1 and Hcp2 together form a tube of stacked Hcp hexamers, which promotes the secretion of effector proteins [23]. Deletion of *hcp1* and *hcp2* gene can affect bacterial adhesion and invasion [24]. In addition, Hcp1 can be secreted by T6SS as an effector in *E. coli*, which promotes cytoskeleton rearrangement and apoptosis of HBMECs. Hcp2 is not detected in the supernatant of bacteria culture, but the deletion of *hcp2* can reduce the expression of bacterial virulence factors, which affects the adhesion and invasion ability of bacteria [23].

Avian pathogenic *E. coli* (APEC) belongs to the extra-intestinal pathogenic *E. coli* (ExPEC). As public health safety has been paid increased attention to globally, the potential zoonotic risks of APEC have also been examined. Several studies have shown the phylogenetic similarity between APEC and NMEC and potential pathogenicity of APEC [23,25,26,27]. APEC had a variety of meningitis-related virulence genes, such as *ibeA*, outer membrane protein T (*ompT*), *ompA* and T6SS [28,29,30,31,32,33,34]. T6SS in the APEC strain has effects on biofilm formation, cell adhesion, virulence factor expression and animal colonization [35,36,37]. Previous studies have pointed out that several APEC strains of serotype O18 could induce meningitis in newborn rats. Although the APEC O78 strain can also be isolated in a small amount of rat cerebrospinal fluid (CSF), morbidity and mortality are far lower than for O18 [25], indicating that different serotypes of APEC have different zoonotic risks.

At present, the main prevalent serotypes of APEC are O1, O2 and O78, with the latter two constituting about 80% of the cases of clinical isolates [38]. DE205B belongs to the O2:K1 serotype, which is a highly virulent strain isolated from the brain of a duck with neural symptoms [39]. Previous studies indicated that DE205B had IbeA, IbeB, AatB and other virulence factors, and had an important role in promoting the pathogenic process of ducks and DF-1 cells [40,41,42]. Whether O2 serotype APEC strain can cause meningitis in mammals or the potential risk of zoonotic disease has not been reported. We chose rats as experimental animals to construct the mammalian model of *E. coli* meningitis [25,43]. In our current research, in order to determine whether DE205B can cause meningitis in rats and evaluate its zoonotic potential, we analyzed the genetic relationship between the DE205B and RS218, and investigated the injury in a mammalian model rat and a cell model HBMEC caused by a DE205B infection. This analysis provided a new understanding of DE205B in the pathogenesis of rat meningitis.

## 2. Materials and Methods

### 2.1. Bacteria, Plasmids and Cell Line

The bacteria and plasmids used in this study are listed in Table 1. The APEC strain DE205B (O2:K1) was isolated from the brain of a duck with neural symptoms and preserved in our laboratory. All bacteria were grown in Luria-Bertani (LB) media or on LB agar plates at 37 °C. Before conducting experiments, bacteria were sub-cultured (1:100) in fresh media.

HBMECs were cultured in high glucose Dulbecco’s Modified Eagle’s Medium (DMEM, Gibco, Cat# C11995500BT) supplemented with 10% heat-inactivated fetal bovine serum (FBS, Gibco, Cat# 10270-106), and 1% (*v*/*v*) penicillin/streptomycin (Solarbio, Cat# P1400) at 37 °C with 5% CO_2_. At the beginning of the experiments, HBMECs were cultured in DMEM without FBS and penicillin/streptomycin at 37 °C with 5% CO_2_.

### 2.2. Phylogenetic Analysis

Thirty- six pathogenic *E. coli* strains (Table 2) with complete genomes from the NCBI database were selected for phylogenetic analysis. Whole genome sequences of 36 *E. coli* were aligned using Parsnp v1.2 from Harvest Suite to obtain core genome single nucleotide polymorphisms (SNPs) (https://www.scopus.com/record/display.uri?eid=2-s2.084965190732&origin=inward&txGid=41b18f9b6b67c71e845056c369278e72, accessed on 23 January 2022) using settings to exclude SNPs identified in regions that had arisen by recombination. The core genome SNPs were used to construct a maximum likelihood phylogenetic tree. The core genome phylogenetic trees were midpoint rooted and annotated using iTOL v6 (https://academic.oup.com/nar/article/47/W1/W256/5424068?login=true, accessed on 23 January 2022).

### 2.3. Construction of Gene Deletion and Complementation Mutants

As previously described, the deletion mutants *Δhcp1* and *Δhcp2* were constructed by the red homologous recombination method [44]. Briefly, pKD4 was used as a template to synthesize PCR target fragment product. The pKD46 plasmid was transferred into the target strain, and the competent state of the target strain was prepared. L-arabinose was added to induce the expression of recombinant enzyme, and the target fragment was electrically transformed into the target strain. The Kana resistance plate was cultured, and the positive strains were screened. The thermosensitive plasmid pCP20 was electrically transformed into the positive target strain to remove the Kana-resistance gene, and the single colonies without Kana-resistance were selected and cultured at 42 °C for more than 24 h to remove the ampicillin resistance. Finally, the nonresistant strain was selected for identification. The construction of the complementation strains *C-Δhcp1* and *C-Δhcp2* were completed by transforming the pSTV28 vector linked to the target genes *hcp1* and *hcp2* into the deleted strains as described in our previous research [45]. Briefly, the wild-type strain was used as a template to amplify the target fragment, ligated into the pSTV28 vector, and the recombinant vector was electrically transformed into the deleted strain, and screened positive strains are identified. The identified primers are shown in Table 3.

### 2.4. Animal Infections

The experimental animals used in this study were all SPF-grade female Sprague-Dawley (SD) rats (from Sipeifu, Beijing, China). The rats were 7 weeks old and weighed 180–200 g at the time of experiments.

DE205B and RS218 were cultured in LB medium to logarithmic phase, then washed twice with sterile PBS, and finally adjusted to 1.0 × 10^8^ CFU/mL with normal saline. The rats were randomly divided into three groups. Each group was inoculated with 5.0×10^7^ CFU/rat of RS218, Wild type (WT) strain DE205B, mutant strain *Δhcp2*, complementation strain *C-Δhcp2*, or PBS by caudal vein injection. Clinical symptoms and body weight of rats in the three groups were monitored within 24 h post infection. Xylazine hydrochloride (2 mg/kg) was administered into the tail vein. The rats were prone after deep anesthesia to collected CSF or brain tissue. For brain tissue, the rats were sacrificed by executing bloodletting, and the brain tissue was collected for pathological section RNA isolation or ELISA.

### 2.5. Isolation and Identification of Bacteria

Twenty-four hours after being injected, the CSF of the rats was aseptically collected through the cisterna cerebello-medullaries using capillary tubes and streaked onto Eosin-Methylene Blue (EMB) plates, cultured overnight at 37 °C to detect the presence of bacteria by Gram staining. The virulence genes of *ibeA*, *icmF*, *ompA*, *fimH*, *hcp1*, *hcp2*, and *CUS-3* were detected by PCR, performed to identify the bacteria isolated from the rats’ CSF. PCR reactions were performed in 20 µL volume, containing 12.5 µL of Green Taq Mix (Vazyme, Cat# P131-01), 1 µL of forward primer, 1 µL of reverse primer, 2 µL of target DNA, and 8.5 µL of ddH_2_O. The amplification program was: 5 min at 95 °C for initial denaturing, and 30 cycles of 40 s at 95 °C, 30 s at 55 °C and 1 min at 72 °C; 10 min at 72 °C for extension. The PCR products were electrophoresed in 1% agar gel. Primers used to identify DE205B are listed in Table 3.

### 2.6. Histopathological Examination and Immunohistochemistry

The pathological changes in the brain of infected rats were also investigated by pathological section and immunohistochemistry. The brain tissues of the control and infected group were collected and fixed in 4% paraformaldehyde solution, embedded in paraffin for tissue sections, stained with HE dyes and finally examined under microscope as previously described [46]. For fluorescence immunohistochemistry, tissue sections of brain were subjected to heat-mediated antigen retrieval for 15 min at 95 °C, and then blocked with 5% BSA in PBS for 1 h at room temperature followed by incubation with primary antibody at 4 °C overnight. After being rinsed in PBS, sections were incubated with Alexa 488- or 647-conjugated secondary antibodies prior to the final nucleus staining with DAPI. Finally, stained sections were photographed on fluorescent inverted microscope. The fluorescent area of GFAP and MPO was analyzed by ImageJ. The following primary antibodies were used: anti-GFAP (GB11096) and anti-MPO (GB11224) purchased from Servicebio, Goat Anti-Mouse IgG (ab150113) and Goat Anti-Rabbit IgG (ab150079) purchased from Abcam.

### 2.7. RNA Isolation of Brain Tissue and RT-PCR

RNA extraction from rat brain tissue was conducted according to the instructions (TaKaRa, Cat# D9108A). In summary, 0.1 g of fresh brain tissue was weighed in a mortar, liquid nitrogen added, brain tissue ground to a powder, 1 mL RNAiso Plus added, centrifuged at 12,000× *g* at 4 °C for 5 min, 200 µL chloroform added to the supernatant, shaken, emulsification of the solution, centrifuged at 12,000× *g* at 4 °C for 15 min, removal of the upper liquid, the same volume of isopropyl alcohol added, centrifuged at 12,000× *g* at 4 °C for 10 min, the supernatant discarded, 1 mL of 75% ethanol added to wash and precipitate, and RNase-Free water used to dissolve RNA. The RNA was reverse transcribed into cDNA using HiScript II 1st Strand cDNA Synthesis Kit (Vazyme, Cat# R211-01). The ΔΔCT method and expression of the housekeeping gene GAPDH was used to determine the purpose gene expression level. The primers for qRT-PCR are listed in Table 3.

### 2.8. Determining the Concentration of Inflammatory Factors

The concentration of inflammatory factors, including IL-6, IL-10 and CXCL-1 in rat brain tissue, were determined by ELISA kits (mlbio, Shanghai, China). One hundred milligrams of fresh brain tissue was homogenized with 1 mL of PBS and the supernatants were used for detection according to the manufacturer’s instructions.

### 2.9. Growth Curve

Growth curves were measured in LB medium. The strains DE205B, *Δhcp1*, *Δhcp2*, *C-Δhcp1* and *C-Δhcp2* were transferred to 20 mL fresh LB medium at a ratio of 1:100 to detect the growth by using automatic biological growth detection reactor RTS1 (BioSan, Riga, Latvia). The growth curve was determined by at least three independent experiments.

### 2.10. HBMECs Invasion Assays

HBMECs cells were grown to confluence in 24-well plates (about 10^5^ cells per well) as described in [47]. Briefly, bacteria were added to confluent HBMECs with multiplicity of infection (MOI) of 100. The plates were incubated for 1.5 h at 37 °C in 5% CO_2_ to allow bacterial invasion to occur. The monolayers were washed three times with sterile PBS and incubated with DMEM containing gentamycin (100 mg/mL) for 1 h. After killing extracellular bacteria and washing, the monolayers were lysed with 0.5% Triton X-100 solution, and the intracellular bacteria were calculated by plates counting.

### 2.11. Immunofluorescence Assays

HBMECs cells were grown to confluence in a confocal dish at 37 °C with 5% CO_2_ overnight as described in [48]. In brief, the monolayers were washed three times with sterile PBS and added bacteria with MOI of 10. The mixtures were incubated for 3 h at 37 °C with 5% CO_2_. Samples were fixed at time points of 1 h, 2 h and 3 h at room temperature, stained by Phalloidin-iFluor 488 (Abcam, Cat# 176753) and incubated for 1 h at room temperature without light, then stained with DAPI for 10 min. Cells in the confocal dish were observed with an inverted fluorescence microscope (ZEISS, Oberkochen, Germany).

### 2.12. Statistical Analysis

Prism 9.0.2 (GraphPad, San Diego, CA, USA) was used to compare the data from the two groups by the two-tailed unpaired Student’s *t* test. Statistical significance was set at *p* < 0.05. For all bar graphs, the mean ± SEM was plotted. Unless otherwise specified, all in vivo and in vitro experiments were independently repeated at least three times.

### 2.13. Ethics Statement

All rats were housed individually in specific pathogen-free cages in the Laboratory Animal Center of Nanjing Agricultural University. The animal study protocol was approved by the Animal Ethics Committee at Nanjing Agricultural University, protocol number SYXK(SU)2011-0036, Nanjing, China. All experiments were performed according to the International Code of Practice for the care and use of animals for scientific purposes. Temperature and humidity were maintained at 21–23 °C and 60–65%, respectively.

## 3. Results

### 3.1. APEC Strain DE205B Genetic Relationship with NMEC Strain RS218

**Figure 1:**
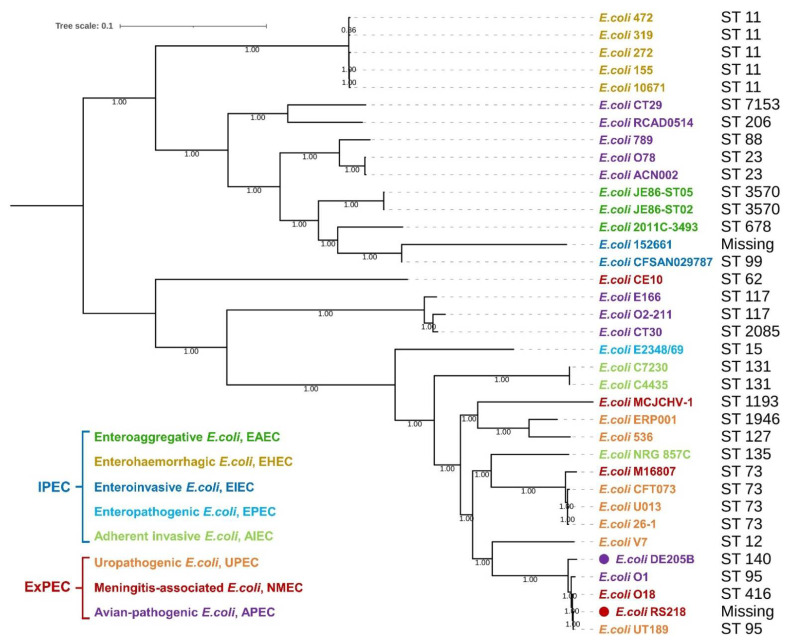
Phylogenetic tree analysis of *E. coli*. The phylogenetic tree of 36 pathogenic *E. coli* strains was analyzed based on the genomes. Numbers given at the nodes represent bootstrap values. The APEC DE205B strain and NMEC RS218 strain were labeled in purple and red circles.

We conducted a phylogenetic tree analysis of 36 different *E. coli* strains based on whole genome sequence of the strains, including 14 intestinal pathogenic *E. coli* (IPEC) and 22 ExPEC strains. The results showed that APEC strains were distributed in different clades. APEC DE205B (O2:K1) clusters with NMEC strains and UPEC strains, including *E. coli* O18, *E. coli* RS218, and *E. coli* UT189, are usually Homo sapiens pathogenic (Figure 1). Similar to RS218, DE205B harbored several virulence genes related to meningitis, including *ibeA*, *icmF*, *ompA*, *fimH*, *hcp1*, *hcp2*, and *CUS-3* (Figure S1).

### 3.2. DE205B Crossed the BBB and Entered the CSF of Rats

**Figure 2:**
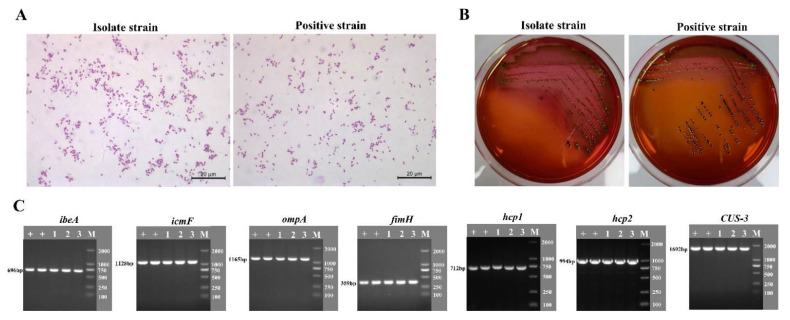
Isolation and identification of DE205B from CSF of rats. Twenty-four hours after receiving the DE205B or RS218 caudal vein infection, CSF of rats was collected from the cerebellomedullary cistern and bacteria were isolated and identified. (**A**) Gram staining of the isolated bacteria. (**B**) Single colony morphology formed on EMB medium. (**C**) Virulence factors identification by PCR. “+” represents the DE205B wild-type strain, “1–3” represents the DE205B isolate, and “M” represents the DNA marker, DL-2000.

The BBB is an important protective barrier of the nervous system. Bacteria crossing the BBB is the prerequisite for meningitis [4]. In order to determine whether DE205B can cross the BBB in mammals, SD rats were used as animal models to infect with DE205B.The CSF of rats was collected 24 h after infection. The results showed that bacteria isolated from CSF of rats were Gram negative rod-shaped by Gram staining (Figure 2A), and formed a single black colony with metallic luster on EMB plates (Figure 2B). The isolated bacteria were further verified as DE205B based on the positive results of the detection of the virulence factors of DE205B by PCR (Figure 2C), indicating that DE205B could enter rat brain tissue through the BBB. No pathogenic bacteria were isolated from the CSF of the control group (data not shown).

### 3.3. DE205B Caused Meningitis in Rats

**Figure 3:**
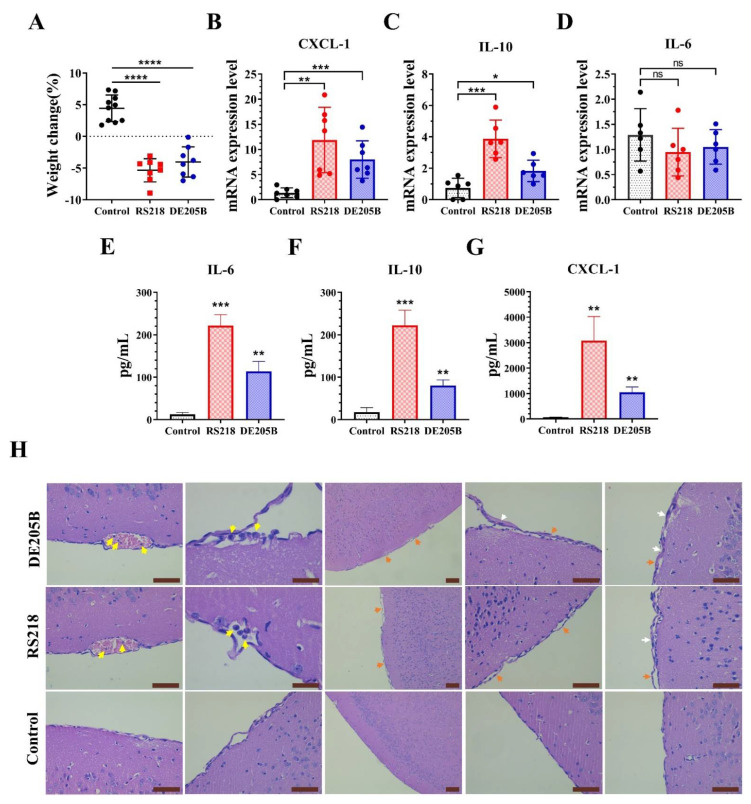
Detection of meningitis in the rat model. Rats received DE205B or RS218 tail vein infection (infection groups) or equivalent amount of PBS (control group). All animals were sacrificed immediately after infection for 24 h. (**A**) Weight change 24 h post infection in rats (*n* = 8–10). The transcriptional levels of CXCL-1 (**B**), IL-10 (**C**) and IL-6 (**D**) in brain tissues of each group (*n* = 6–7). The content of IL-6 (**E**), IL-10 (**F**), CXCL-1 (**G**) in each 100 mg brain tissue was detected by ELISA. (**H**) Representative images of brain tissue sections from rats infected with DE205B, RS218, and PBS. The yellow arrows indicate neutrophils, the orange arrows indicate detachment of the meninges, and the white arrows indicate thickening of the meninges. Scale bars denote 200 μm. Groups were compared by unpaired *t*-tests, * *p* < 0.05, ** *p* < 0.01, *** *p* < 0.001, **** *p* < 0.0001, ns denotes no significant difference, the centerline indicates mean values, and error bars denote the standard deviations.

We have established that the APEC strain DE205B could enter the brain tissue of rats. Next, we tried to study the effect of DE205B on the brain tissue. In order to determine the inflammation level of brain tissue mediated by DE205B, RT-PCR analysis was performed on the brain tissue of infected rats. The results showed that the transcription levels of chemokine CXCL-1 (*p* < 0.001) and inflammatory factor IL-10 (*p* < 0.05) were increased in the brain tissue of the infected group compared to the control group (Figure 3B,C). Compared with the control group, CXCL-1 in the RS218 infection group and DE205B infection group increased 8.62-fold and 5.80-fold and IL-10 increased 5.19-fold and 2.46-fold, respectively. There was no significant change in transcription levels of inflammatory factor IL-6 in brain tissue of rats infected with RS218 and DE205B (Figure 3D). The concentration of inflammation factors, including IL-6, IL-10, CXCL-1 in the brain tissue was further detected by ELISA. Consistent with RT-PCR results, compared to the control group, the concentration of CXCL-1 and IL-10 were significantly increased in brain tissue of RS218 (*p* < 0.01, *p* < 0.001) and DE205B (*p* < 0.01, *p* < 0.01) infection groups (Figure 3F,G). Although the transcript level of IL-6 was not obviously changed, the concentration of IL-6 in the brain was significantly increased in the RS218 (*p* < 0.001) and DE205B (*p* < 0.01) infected groups (Figure 3E). Pathological analysis of brain tissue showed that neutrophil infiltration, meningeal detachment, and thickening were observed in the DE205B infection group, which was similar to that in the RS218 infection group (Figure 3E). Moreover, the weight of rats in the DE205B infection group decreased 24 h after infection (*p* < 0.001, Figure 3A).

### 3.4. The Effect of DE205B on the Nervous System

**Figure 4:**
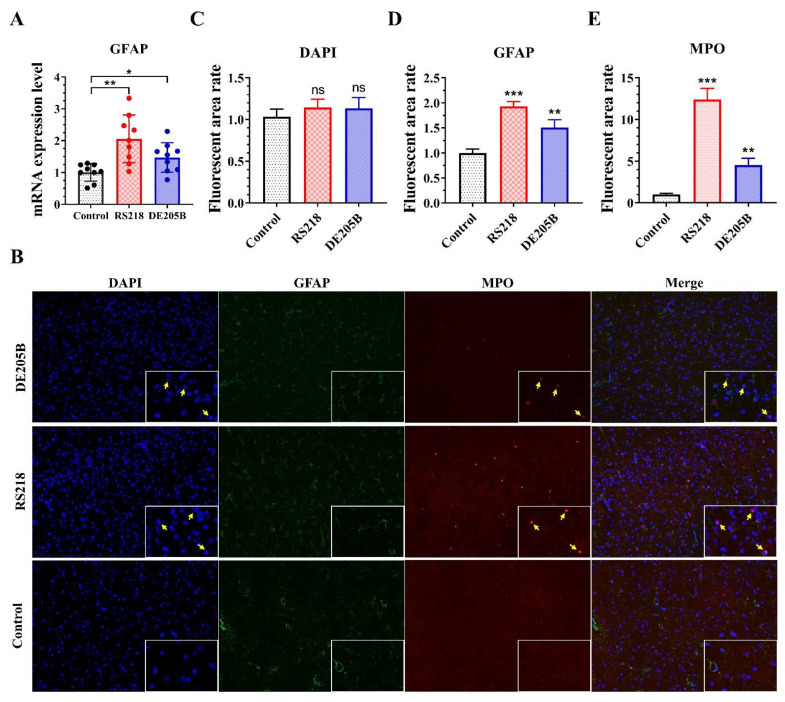
Detection of nerve injury in rat brain tissue. (**A**) The transcription level of GFAP in rat brain tissue (*n* = 9). (**B**) Representative immunohistochemical images of rat brain tissue infected with DE205B, RS218, and normal saline using immunofluorescence against DNA (blue), GFAP (green), and MPO (red). Yellow arrows indicate neutrophils. The magnification was 200×. (**C**–**E**) Quantification of fluorescence in the different groups. ImageJ software was used to analyze the fluorescence area of the immunofluorescence photos, and the fluorescence area rate was the ratio of the infected group to the control group. Groups were compared by unpaired *t*-test, and error bars denotes the standard deviations. * *p* < 0.05, ** *p* < 0.01, *** *p* < 0.0001, ns denotes no significant difference.

To determine whether meningitis caused by DE205B infection caused neurological damage, we detected for glial fibrillary acidic protein (GFAP), a marker of nerve damage, in brain tissue [49]. The results showed that the transcription level of GFAP increased in the DE205B infection group (*p* < 0.05), compared to levels observed in the RS218 (*p* < 0.01) infection group (Figure 4A). Correspondingly, the protein expression level of GFAP in the hippocampus of rats also increased (Figure 4B,D). In addition, infiltration of neutrophils in the brain parenchyma of the DE205B and RS218 infection groups was observed (Figure 4B,E). Results indicated that DE205B could not only cause meningeal inflammation, but also caused damage to the rat’s nervous system. This process may be related to the inflammation caused by neutrophils infiltrating the brain parenchyma.

### 3.5. Hcp1 and Hcp2 Jointly Promoted the Invasion of HBMECs

**Figure 5:**
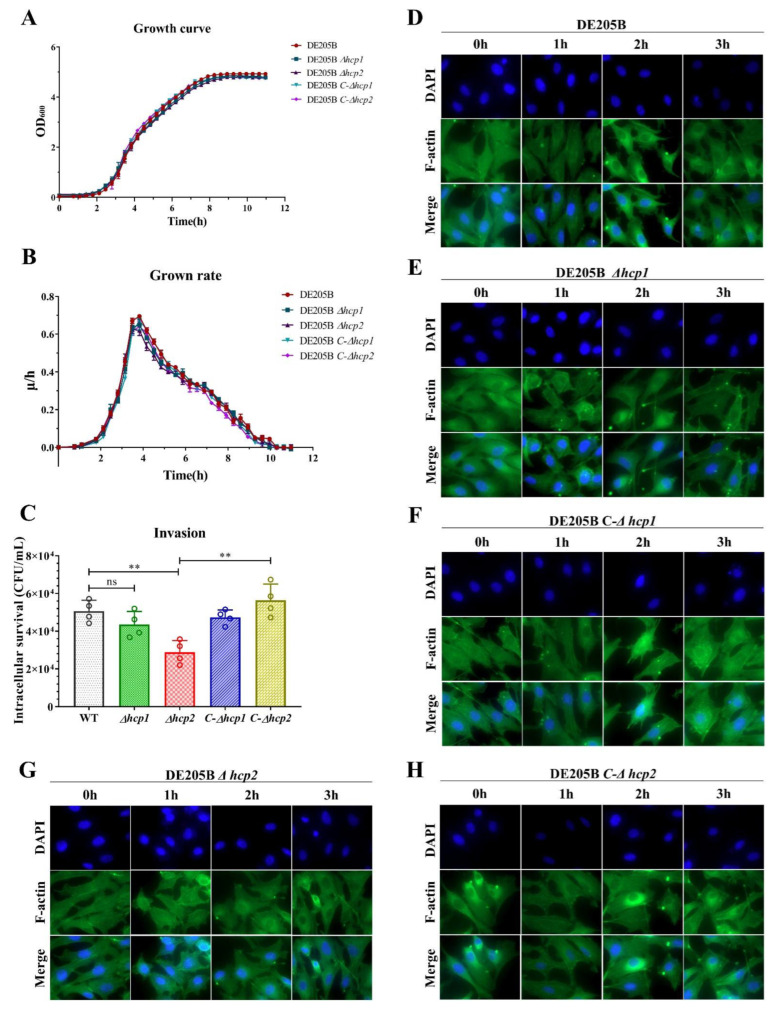
Effects of the invasion and cytoskeleton rearrangement of HBMECs infected wild-type and deletion strains of DE205B. Growth curves (**A**) and growth rates (**B**) of wild-type, deletion and complementation strains at OD_600_ nm. (**C**) Invasion assays. HBMECs were incubated with wild-type, deletion or complementation strains for 1.5 h with an MOI of 100. (**D**–**H**) Assessment of cytoskeleton rearrangement. HBMECs were incubated with strain WT, *Δhcp1*, *C-Δhcp1*, *Δhcp2*, or *C-Δhcp2* for 0 h, 1 h, 2 h, and 3 h, respectively. Cytoskeleton rearrangements were observed by fluorescence change of F-actin (green). DNA (blue) was stained with DAPI and magnification was 600×. Groups were compared by unpaired *t*-test, and error bars indicate the standard deviations. ** *p* < 0.01, ns denotes no significant difference.

The adhesion and invasion of BMECs by *E. coli* is a key step for *E. coli* in penetrating the BBB and entering the brain tissue to mediate *E. coli* meningitis [4]. However, the mechanism by which DE205B invades BMECs to cross the BBB remains unclear. We found that DE205B had a complete T6SS, in which the Hcp1 and Hcp2 proteins had high homology with RS218 (Figure S2). In order to study the role of two Hcp proteins in the process of DE205B adhesion and invasion of BMECs, we used HBMECs as an in vitro infection model. *hcp1* and *hcp2* deletion and complementation mutants of DE205B were constructed. It was confirmed that the two genes had no effect on the growth of DE205B (Figure 5A,B) and the results of the HBMECs invasion test showed that the intracellular survival number of strains WT and *Δhcp2* were 5.06 × 10^4^ CFU/mL and 2.89 × 10^4^ CFU/mL, respectively, indicating that the invasion ability of the *Δhcp2* strain to HBMECs was significantly decreased compared to the WT (*p* < 0.01), and the invasion ability of the *C-Δhcp2* strain was recovered (*p* < 0.01), while the *Δhcp1* strain had no significant change (*p* ≥ 0.05) (Figure 5C). These results indicated that Hcp2 protein of DE205B was involved in the invasion of HBMEC, and Hcp1 had no effect in the process.

Because cytoskeleton rearrangement of host cells is related to bacterial invasion of HBMECs, we tested whether DE205B strain induced HBMEC cytoskeleton rearrangement to promote bacterial invasion. With the extension of time, stress fibers in HBMECs gradually formed, which induced the gradual rearrangement of cytoskeleton, and HBMECs gradually changed from fusiform to flat shape (Figure 5D). However, after the deletion of *hcp1* gene, no obvious formation of stress fiber was observed over time (Figure 5E). The phenotype was recovered in the *C-Δhcp1* strain (Figure 5F). In contrast, the Hcp2 protein had no significant effect on cytoskeletal rearrangement of HBMECs (Figure 5G,H).

### 3.6. Protein Hcp2 Was Conducive to DE205B Invading Brain Tissue and Aggravated Inflammatory Response

**Figure 6:**
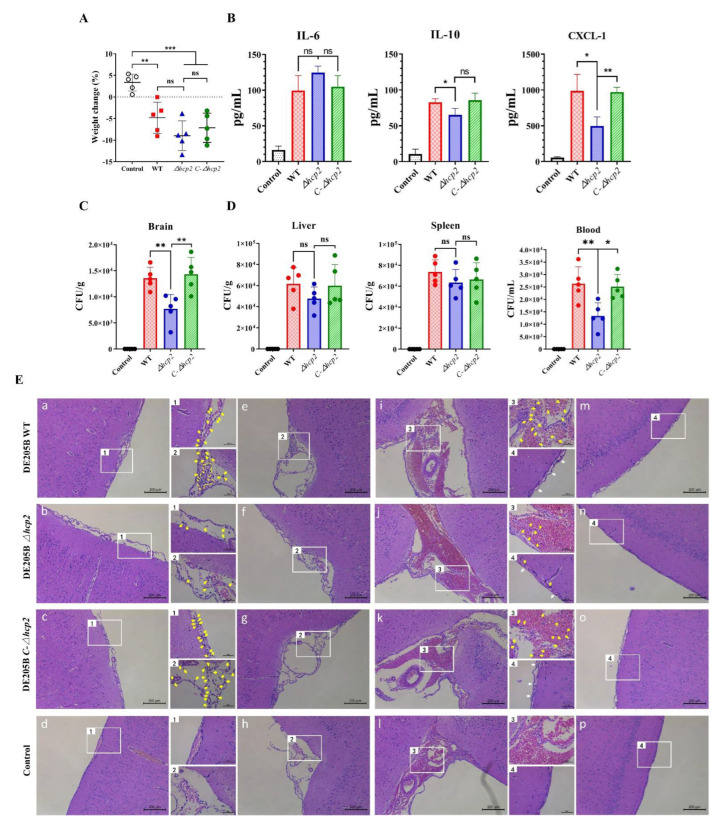
Detection of the ability of strain *Δhcp2* to induce meningitis in rats. Rats received WT, *Δhcp2*, or *C-Δhcp2* strains tail vein infection (infection groups) or equivalent amount of PBS (blank group). (**A**) Weight change 24 h post infection in rats (*n* = 5). (**B**) Contents of inflammatory factors in brain tissue of different infection groups, including IL-6, IL-10, CXCL-1. (**C**, **D**) Bacterial contents in brain, liver, spleen, and blood of different infection groups. (**E**) Representative images of brain tissue sections from rats infected with strain WT, *Δhcp2*, *C-Δhcp2*, or PBS. The yellow arrows indicate neutrophils, and the white arrows indicate thickening and separation of the meninges. Scale bars denote 200 μm. Groups were compared by unpaired *t*-tests, * *p* < 0.05, ** *p* < 0.01, *** *p* < 0.001, ns denotes no significant difference, the centerline indicates mean values, and error bars denote the standard deviations.

To prove whether Hcp2 affects the ability of DE205B to induce meningitis, rats were injected with WT strain DE205B, mutant strain *Δhcp2*, or complementation strain *C-Δhcp2* as the infection groups and PBS as the blank group. There was no significant difference in body weight changes between WT and *Δhcp2 group* (Figure 6A). However, compared with WT group, the levels of IL-10 (*p* < 0.05) and CXCL-1 (*p* < 0.05) in the brain tissue of rats infected with *Δhcp2* strain were significantly reduced, among which CXCL-1 was most reduced, and the level of IL-10 and CXCL-1 were recovered in *C-Δhcp2* group (Figure 6B). There was no significant change in the level of IL-6 between the WT and *Δhcp2* group (Figure 6B). Subsequently, 24 h after infection, we detected the bacterial load in the brain tissue of different groups. Compared with the WT group, the bacterial load in brain tissue of *Δhcp2* group was significantly reduced (*p* < 0.01, Figure 6C). The bacterial load was recovered in *C-Δhcp2* group (*p* < 0.01, Figure 6C), indicating that the ability of *Δhcp2* strain to penetrate the BBB and invade into the brain tissue was weakened, which was consistent with the results of the HBMECs invasion assays. In addition, the content of *Δhcp2* strain in the liver and spleen was not significantly reduced, but the survival ability in blood was reduced to 50.8% (Figure 6D), indicating that Hcp2 not only affected bacterial invasion but also promoted the viability of DE205B in the blood.

The results of the pathological analysis showed that neutrophils were infiltrated in the brain tissue of rats infected with WT strain, *Δhcp2* strain, or *C-Δhcp2* strain, and the number of neutrophils in *Δhcp2* group was significantly lower than that in WT and *C-Δhcp2* group (Figure 6E(a–d)). To avoid the influence of subjective preference on the result, we selected the same area of brain tissue for imaging analysis and reached the same conclusion (Figure 6E(e–l)). This showed that the deletion of the *hcp2* gene reduced the amount of DE205B invading brain tissue, resulting in a lower level of inflammatory response (Figure 6B) and alleviating pathological changes in meningitis, such as meningeal thickening and separation (Figure 6E(m–p)). The reduction in the number of invasions of the *Δhcp2* strains in vitro corresponded to the results of meningitis in rat models. These results re-emphasized the importance of Hcp2 in DE205B induced meningitis.

The above results suggested that the Hcp1 and Hcp2 proteins of DE205B played different roles in the invasion of HBMECs. Hcp1 mainly induced cytoskeleton rearrangement, while Hcp2 contributed to the survival of DE205B in the blood and the invasion of brain tissue, aggravating the inflammatory response of brain tissue. Hcp1 and Hcp2 synergistically promoted DE205B across the BBB to cause meningitis.

## 4. Discussion

Public health and the prevention of zoonotic infectious diseases have received increasing attention. Bacterial meningitis is an important concern. Due to the immature immune system of newborns, NMEC infection often occurs during childbirth [50]. Although we have made progress in antimicrobial treatment and prevention, the widespread use of antibiotics has increased the antibiotic resistance of many pathogenic bacteria, leading to ineffective clinical treatment results [51,52]. Therefore, the prevention of ExPEC infection is an important solution, which requires us to further understand the source and pathogenic mechanism and prevent the spread of pathogenic bacteria from the root. Poultry is the main source of human ExPEC infection, and APEC has a high homology with human ExPEC, in which APEC and NMEC share some common virulence factors and phenotypes [38,53]. These virulence factors have been reported to be related to meningitis caused by NMEC strain RS218, suggesting that APEC may also cause mammalian meningitis through these virulence factors [24,25,27,29].

O1, O2 and O78 are now the most frequently observed serotypes of APEC. Previous research mentioned a variety of APEC O18 strains, which caused meningitis in neonatal rats [25]. However, the pathogenicity of APEC O2 serotype, one of the more frequently observed serotypes, in causing mammalian meningitis disease is still unclear, and its zoonotic risk is unknown. Here, we analyzed the previously isolated APEC strain, DE205B (O2:K1) from duck brain tissue. In the phylogenetic tree analysis, we found that DE205B only had similar genetic similarity with RS218, but not the NMEC O18, CE10, M16807, and HCJCHV-1 strains. DE205B not only contained K1 capsule, but also contained RS218’s unique prophage gene CUS-3, which is in line with the high homology between APEC and human ExPEC reported previously, indicating that DE205B may have RS218 similar pathogenicity in mammalian infections, and is a potential zoonotic pathogen.

Although patients with bacterial meningitis can be cured, 20–50% of patients still have severe and permanent neurological sequelae, which indicates that inflammation of brain tissue and pathogenic bacteria and their products can cause brain damage. The level of inflammatory response to bacterial infection determines the degree of brain damage caused by bacterial meningitis [3,54,55]. Surprisingly, there was no significant change in transcription levels of inflammatory factor IL-6 in brain tissue of rats infected with RS218 and DE205B. The reason for this phenomenon may be that there are processes such as post-transcriptional processing, degradation, translation, post-translational processing, and modification in the transcription and translation of eukaryotic gene expression, so the level of transcription and translation may not be completely consistent. Neutrophils are the forerunner of bacterial infection-induced inflammation and an important member of autoimmunity [56]. However, excessive inflammation in bacterial meningitis can cause central nervous system damage. IL-1β, TNF-α, reactive oxygen species (ROS) and matrix metalloproteinases (MMPs) are released into CSF, which promotes the destruction of BBB integrity, and even had direct toxic effects on neurons [57,58,59]. The excessive recruitment of neutrophils into the brain tissue can lead to more severe meningeal inflammation. On the contrary, knocking-out neutrophils can effectively reduce the occurrence of *E. coli* meningitis in newborn mice [16], In this experiment, we have detected neutrophil infiltration and neuronal damage in the meninges and brain parenchyma of rats infected with DE205B and RS218, indicating that the excessive infiltration of neutrophils may be one of the main causes of nervous system damage in *E. coli* meningitis, which was consistent with the results of previous studies [16]. Therefore, reducing the level of brain inflammation by alleviating the infiltration of neutrophils and the release of inflammatory factors is one of the directions to prevent and treat the neurological sequelae of *E. coli* meningitis.

T6SS is ubiquitous in Gram-negative bacteria, and is often associated with the pathogenicity of many bacteria, including *Vibrio cholerae*, *Legionella pneumophila*, *Aeromonas hydrophila*, *Pseudomonas aeruginosa*, and *Burkholderia pseudomallei* [60,61,62,63]. T6SS in the APEC strain has effects on biofilm formation, cell adhesion, virulence factor expression and animal colonization [36,37]. Here, we confirmed that Hcp1 and Hcp2, two effector proteins in T6SS of DE205B strain, jointly promoted the invasion of HBMECs through different pathways. Hcp proteins are required for both T6SS assembly and effector secretion, and have been identified as chaperones, substrate receptors, and secretory proteins [64,65]. Hcp protein plays an important role in bacterial pathogenicity. For example, in *B. pseudomallei* from Thailand, Hcp1 mediates bacterial antagonism, while Hcp5 promotes the proliferation of macrophages [66]. Hcp1 and Hcp2B inhibit macrophage phagocytosis in APEC XM strains [31]. Here, we proved that Hcp1 induced HBMECs cytoskeleton rearrangement in DE205B, but the specific mechanism is still unclear. Previous research supported that Hcp1 could be secreted extracellularly or injected into cells through the needle-like system of T6SS [67]. Hcp2 protein is the structural protein of bacterial T6SS. When the *hcp2* gene was deleted, it reduced the invasion of DE205B in HBMECs. Previous studies have shown that the Hcp2 gene was related to the regulation of bacterial virulence factor expression. When the Hcp2 gene was deleted, the expression of virulence factors such as OmpA, FimA, and FimC decreased, which may be related to the survival in the blood and invasion in the brain. Therefore, whether DE205B *Δhcp2* reduces the invasion of the brain tissue due to Hcp2-mediated or through down-regulation of other virulence factors, requires further study.

As an APEC strain, DE205B can cause meningitis and neurological symptoms in ducks [39]. At the same time, it contained various meningitis-related virulence genes of NMEC. It had high genetic similarity with NMEC strain RS218, which made us doubt whether DE205B carries zoonotic risk. Rats are commonly used experimental animals in the study of human diseases, including meningitis [25,43]. In this study, we demonstrated that DE205B can cross the BBB via peripheral blood into brain tissue and mediate a range of pathological characteristics of meningitis in rats, which indicated that it might have particular virulence and pathogenic risk in mammals and was a potential foodborne zoonotic pathogen. The zoonotic risk of APEC has received increasing attention, and multiple APEC serotypes have been reported to cause meningitis in mammals, including O1 and O18 [25,38]. Here we provided evidence for the zoonotic risk of APEC O2 serotypes.

In summary, DE205B (O2:K1), belonging to one of the most frequently observed serotypes of APEC, had similar genetic similarity and virulence factors to RS218. DE205B synergistically promoted the invasion of HBMECs through Hcp1 and Hcp2 proteins of T6SS. In the rat infection models, DE205B crossed the BBB and entered the brain tissue, causing meningitis in rats and causing damage to the nervous system. The deletion of *hcp2* gene can reduce survival ability in the blood and invasive ability in the brain of DE205B, thereby alleviating the process of meningitis.

## Figures and Tables

**Table 1:** Bacterial strains and plasmids used in this study.

Bacterial Strains and Plasmids	Genotype or Relevant Characteristics
Bacterial strains	
RS218	O18:K1:H7 stains from human
DE205B	O2:K1 stains from duck
DE205B *Δhcp1*	*hcp1* gene deletion in DE205B
DE205B *Δhcp2*	*hcp2* gene deletion in DE205B
DE205B *C-Δhcp1*	DE205B *Δhcp1* with plasmid pSTV28-*hcp1*
DE205B *C-Δhcp2*	DE205B *Δhcp2* with plasmid pSTV28-*hcp2*
Plasmids	
pKD4	template for λ-Red Kanr cassette
pKD46	λ-Red recombinase expression
pCP20	encodes FLP recombinase for removal of resistance cassette
pSTV28	A medium-copy plasmid

**Table 2:** Pathotypes and accession numbers of 36 *E. coli* strains.

*E. coli* Strains	Pathotype	Serotype	Host	Year	Geographic Location	GenBank
*E. coli* JE86-ST02	EAEC	O86:H27	Homo sapiens	1999	Japan	AP022811.1
*E. coli* JE86-ST05	EAEC	O86:H27	Homo sapiens	2014	Japan	AP022815.1
*E. coli* 2011C-3493	EAEC	O104:H4	Homo sapiens	2011	USA	CP003289.1
*E. coli* 155	EHEC	O157:H7	Homo sapiens	2012	United Kingdom	CP018237.1
*E. coli* 272	EHEC	O157:H7	Homo sapiens	2013	United Kingdom	CP018239.1
*E. coli* 319	EHEC	O157:H7	Homo sapiens	2012	United Kingdom	CP018241.1
*E. coli* 472	EHEC	O157:H7	Homo sapiens	2012	United Kingdom	CP018245.1
*E. coli* 10671	EHEC	O157:H7	Homo sapiens	2012	United Kingdom	CP018250.1
*E. coli* CFSAN029787	EIEC	O96:H19	Homo sapiens	2012	Italy	CP011416.1
*E. coli* 152661	EIEC	O96:H19	Homo sapiens	2014	United Kingdom	CP046676.1
*E. coli* E2348/69	EPEC	O127:H6	Homo sapiens	2012	United Kingdom	NZ_LT827011.1
*E. coli* NRG 857C	AIEC	O83:H1	Homo sapiens	2008	USA	CP001855.1
*E. coli* C4435	AIEC	O25:H4	Homo sapiens	2010	Mexico	CP027851.1
*E. coli* C7230	AIEC	O25:H4	Homo sapiens	2012	Mexico	NZ_PXXQ01000001.1
*E. coli* UT189	UPEC	Missing	Homo sapiens	2018	India	CP062228.1
*E. coli* V7	UPEC	Missing	Homo sapiens	1981	USA	CP048855.1
*E. coli* 26-1	UPEC	Missing	Homo sapiens	2012	South Korea	CP016497.1
*E. coli* U013	UPEC	Missing	Homo sapiens	2014	China	CP058596.1
*E. coli* ERP001	UPEC	Missing	Red panda	2017	China	CP063214.1
*E.coli* 536	UPEC	O6:K15:H31	Homo sapiens	Missing	Missing	NC_008253.1
*E.coli* CFT073	UPEC	O6:H1	Homo sapiens	1990	USA	NC_004431.1
*E. coli* O18	NMEC	O18:K1	Avian/Homo sapiens	1989	Netherlands	CP007275.1
*E. coli* MCJCHV-1	NMEC	O75:H5:K1	Homo sapiens	2015	USA	NZ-CP030111.1
*E. coli* CE10	NMEC	O7:K1	Homo sapiens	Missing	USA	CP003034.1
*E. coli* M16807	NMEC	Missing	Homo sapiens	Missing	USA	CP031256.1
*E. coli* RS218	NMEC	O18:H7:K1	Homo sapiens	1974	Missing	CP007149.1
*E.coli* DE205B	APEC	O2:K1	Duck	2011	China	This study
*E. coli* O2-211	APEC	O2	Gallus gallus	1982	USA	NZ-CP006834.2
*E. coli* O1	APEC	O1:K1:H7	Turkey	2006	USA	NC-008563.1
*E. coli* O78	APEC	O78	Duck	2017	China	NC-020163.1
*E. coli* RCAD0514	APEC	Missing	Duck	2017	China	NZ-CP034106.1
*E. coli* CT30	APEC	Missing	Avian	2014	China	NZ-CP032078.1
*E. coli* E166	APEC	Missing	Avian	2014	China	NZ-CP032066.1
*E. coli* CT29	APEC	Missing	Avian	2014	China	NZ-CP032073.1
*E. coli* ACN002	APEC	Missing	Avian	2016	China	NZ-CP007491.1
*E. coli* 789	APEC	O78:H19	Turkey	1990	Missing	NZ-CP010315.1

**Table 3:** PCR Primers used in this study.

Gene	Sequence (5′–3′)
For RT-PCR	
GFAP-F	CGTGGAGATGGATGTGGC
GFAP-R	TCTGCAAACTTGGACCGA
CXCL-1-F	GGCAGGGATTCACTTCAAGA
CXCL-1-R	ACTTGGGGACACCCTTTAGC
IL-6-F	CCAGCCAGTTGCCTTCTT
IL-6-R	TCTGTTGTGGGTGGTATCCT
IL-10-F	GCCCAGAAATCAAGGAGCAT
IL-10-R	CGTAGGCTTCTATGCAGTTG
GAPDH-F	ATGGGAAGCTGGTCATCAAC
GAPDH-R	GGATGCAGGGATGATGTTCT
General PCR for cloning	
OmpA-F	TTGGATGATAACGAGGCG
OmpA-R	CAGGCATTGCTGGGTAAG
IcmF-F	GGGTGGCGAAGATTGG
IcmF-R	GCGTAGGGCCGTATGT
FimH-F	GTTATTACCCTGTTTGCTG
FimH-R	GGCTTATCCGTTCTCG
IbeA-F	GAAGTGTTAGTTGTTGGTGGTG
IbeA-R	TCCTGCCGACTTTCCTTT
CUS-3-F	CTTCCCTTCGGCGGTTGT
CUS-3-R	TCCGCTTATGAAAGGTGTCG
For Deletion ^a^	
Hcp1-P1	CGGGAGCAATTTCTTCCTTTACTGACATACTGAATATCCTTCTGTGAAAAgtgtaggctggagctgcttcga
Hcp1-P2	TGTATGCAGTACGAAAATGCTGTGCTCATGGCCTGAACGGGAACATTTTTcatatgaatatcctccttag
Hcp2-P1	GACGGGTTGTTCGTAAAACAGCAGTTGATAATTTCACAAGGAGTTCATAA gtgtaggctggagctgcttcga
Hcp2-P2	ACGTACAAAAACAACATCCTGCACGGAGGCAGGATGTTGTTGACTCAGATcatatgaatatcctccttag
For Complemented ^a^	
Hcp1-F	tatgaccatgattacgaattcTTATTTCTGAACGGCGATACCC
Hcp1-R	cttgcatgcctgcaggtcgacATGAGCAAAATGAACAACAATGGC
Hcp2-F	tatgaccatgattacgaattcATGCCAACCCCATGTTACATTT
Hcp2-R	cttgcatgcctgcaggtcgacTTATGCTTCCAGCGGTGCA
For deletion identification ^b^	
K1	CAGTCATAGCCGAATAGCCT
K2	CGGTGCCCTGAATGAACTGC
Kt	CGGCCACAGTCGATGAATCC
Hcp1-up	TTCTGATTTAGGCTGGACGC
Hcp1-down	CTGCCACTGAAACGGTATTG
Hcp2-up	ACTCTAACCTGTCGGGGATT
Hcp2-down	GTCAGCGTTGCGTTCTTCT

^a^ Capital letters represent homologous fragments of the deleted genes. ^b^ Partial primers are also used in general PCR for cloning.

## Data Availability

Not applicable.

## Supplementary Materials

The following supporting information can be downloaded at: https://www.mdpi.com/article/10.3390/life12091353/s1, Figure S1: Identification of meningitis-related virulence factor by PCR. Lanes 1–2: RS218 positive strains; lanes 3–4: DH5α negative strains; lanes 5–7: DE205B strains; M: DL-2000. The size of the target gene length was mapped; Figure S2: (A,B) DNA sequence and amino acid sequence alignment of *hcp1* and *hcp2* between RS218 strain and DE205B strain. *hcp1* and *hcp2* gene of DE205B shares high sequence homology with RS218. “*” means terminator.
