# Supplementary material for: Hcp Proteins of the Type VI Secretion System Promote Avian Pathogenic E. coli DE205B (O2:K1) to Induce Meningitis in Rats
Source: Life (Basel). 2022 Aug 31;12(9):1353. doi: 10.3390/life12091353 (PMC9503490; doi:10.3390/life12091353)
Supplement: Supplementary file 1 [file life-12-01353-s001.zip › life-1826887-supplementary.pdf]

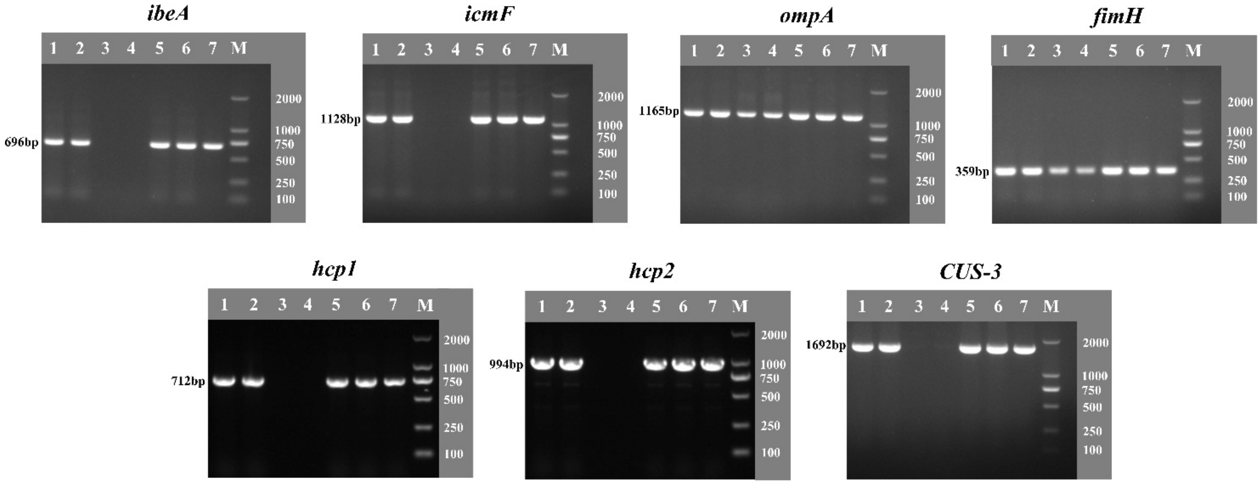

Figure S1. Identification of meningitis-related virulence factor by PCR. Lanes 1–2: RS218 positive strains; lanes 3–4: DH5α negative strains; lanes 5–7: DE205B strains; M: DL-2000. The size of the target gene length was mapped.

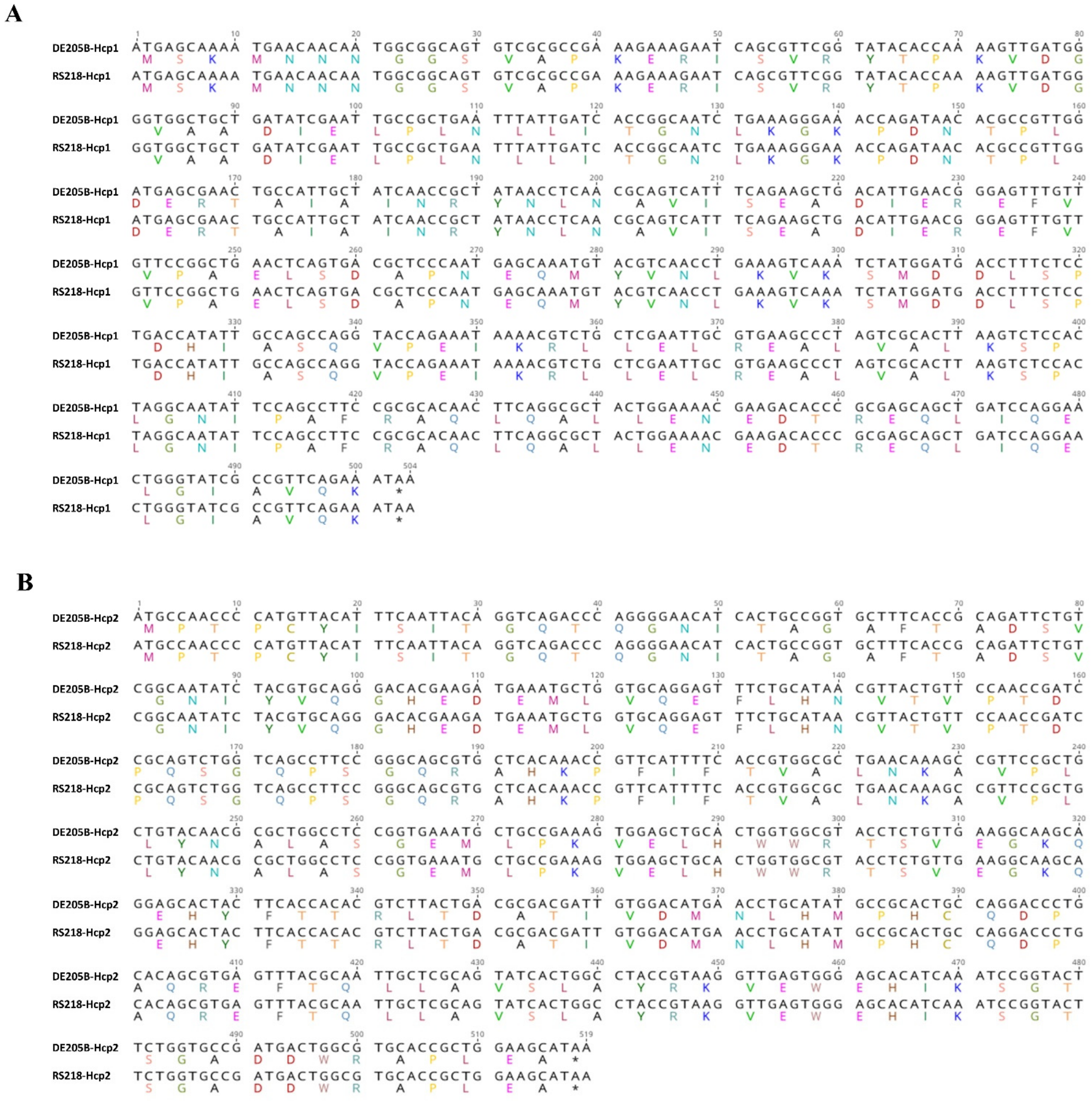

Figure S2. (**A**,**B**) DNA sequence and amino acid sequence alignment of *hcp1* and *hcp2* between RS218 strain and DE205B strain. *hcp1* and *hcp2* gene of DE205B shares high sequence homology with RS218. “*” means terminator.
